# Supplementary material for: A disease-associated gene desert directs macrophage inflammation through ETS2
Source: Nature. 2024 Jun 5;630(8016):447–56. doi: 10.1038/s41586-024-07501-1 (PMC11168933; doi:10.1038/s41586-024-07501-1)
Supplement: Supplementary file 1 — Supplementary Fig. 1: uncropped Western blots from Fig. 2d. Two lanes were run for each sample: one lane to blot for vinculin and the NADPH oxidase components gp91phox, gp65 and p22phox, and one lane to blot for vinculin and the chaperone protein EROS. After transfer, the membranes were cut to blot for individual targets. Supplementary Fig. 2: example gating strategy. Example gating strategy for MPRA and macrophage phenotyping. Macrophages were gated by FSC-A/SSC-A and singlets were gated by FSC-A/FSC-H. Live cells were gated (and viability was quantified) using Live/Dead Fixable Aqua Dead Cell Stain. [file 41586_2024_7501_MOESM1_ESM.pptx]

## Slide 1
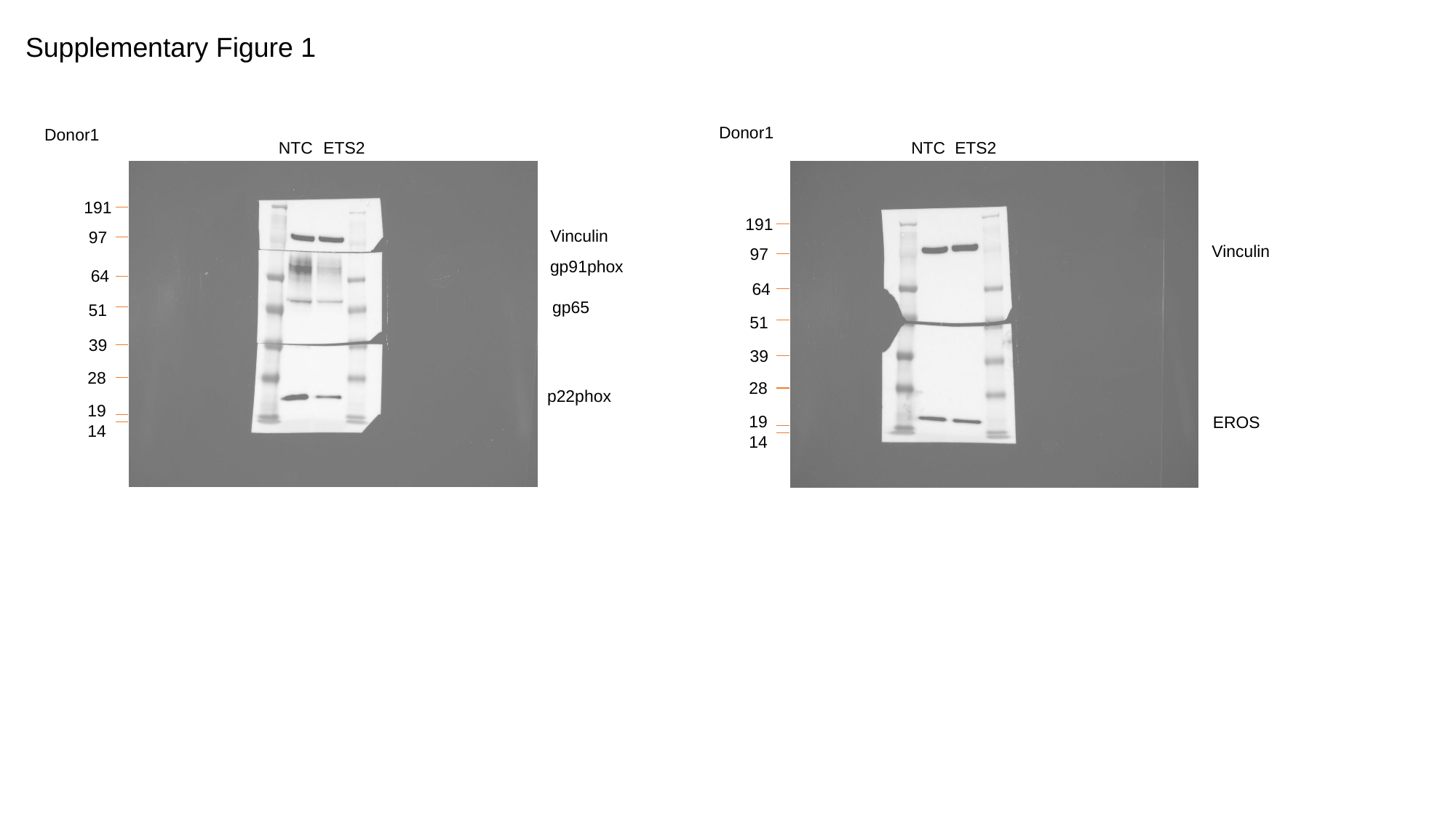

Supplementary Figure 1
Donor1
Donor1
NTC
ETS2
NTC
ETS2
191
191
Vinculin
97
Vinculin
97
gp91phox
64
64
gp65
51
51
39
39
28
28
p22phox
19
19
EROS
14
14

## Slide 2
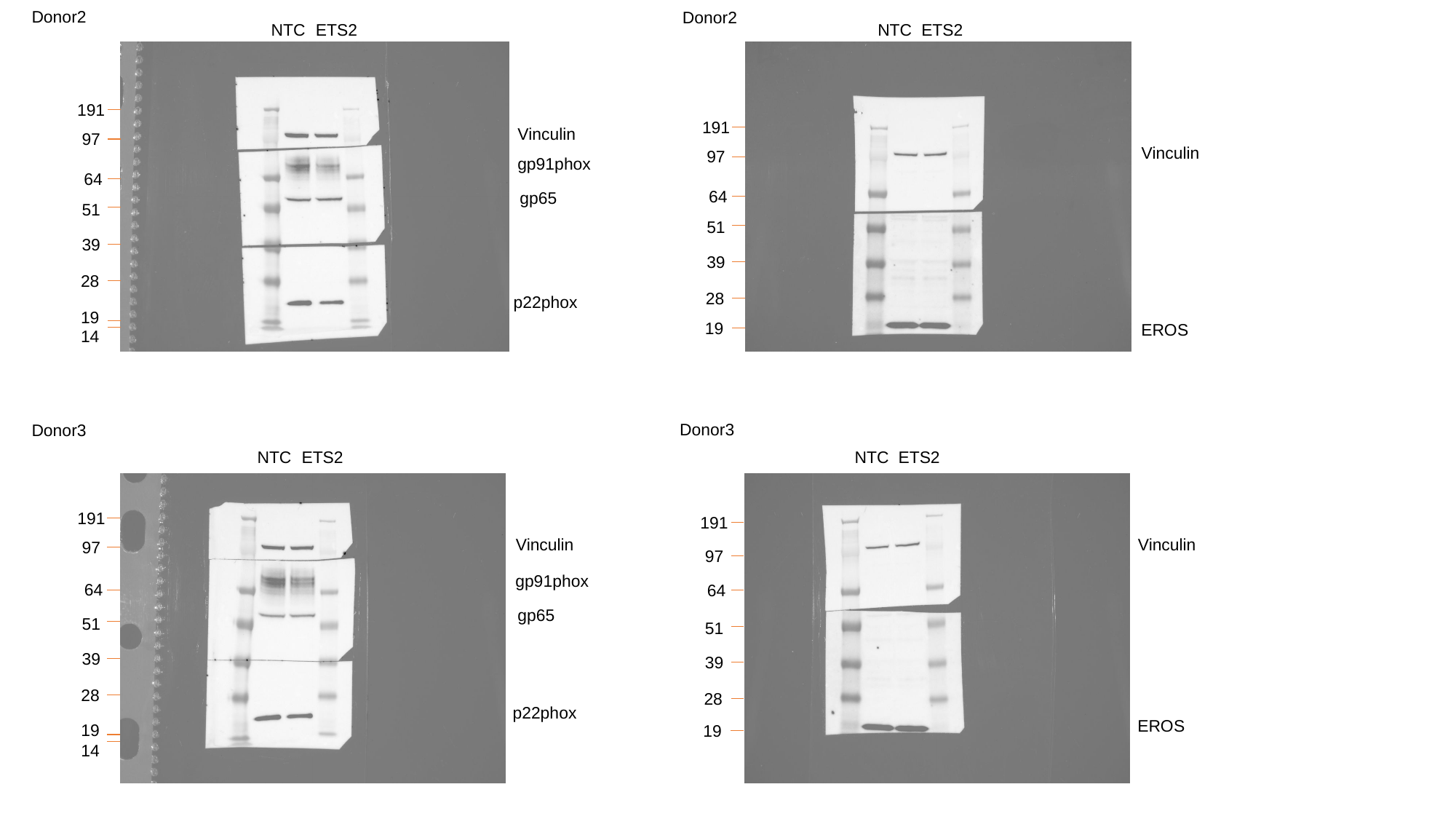

Donor2
Donor2
NTC
ETS2
NTC
ETS2
191
191
Vinculin
97
Vinculin
97
gp91phox
64
64
gp65
51
51
39
39
28
28
p22phox
19
19
EROS
14
Donor3
Donor3
NTC
ETS2
NTC
ETS2
191
191
Vinculin
Vinculin
97
97
gp91phox
64
64
gp65
51
51
39
39
28
28
p22phox
EROS
19
19
14

## Slide 3
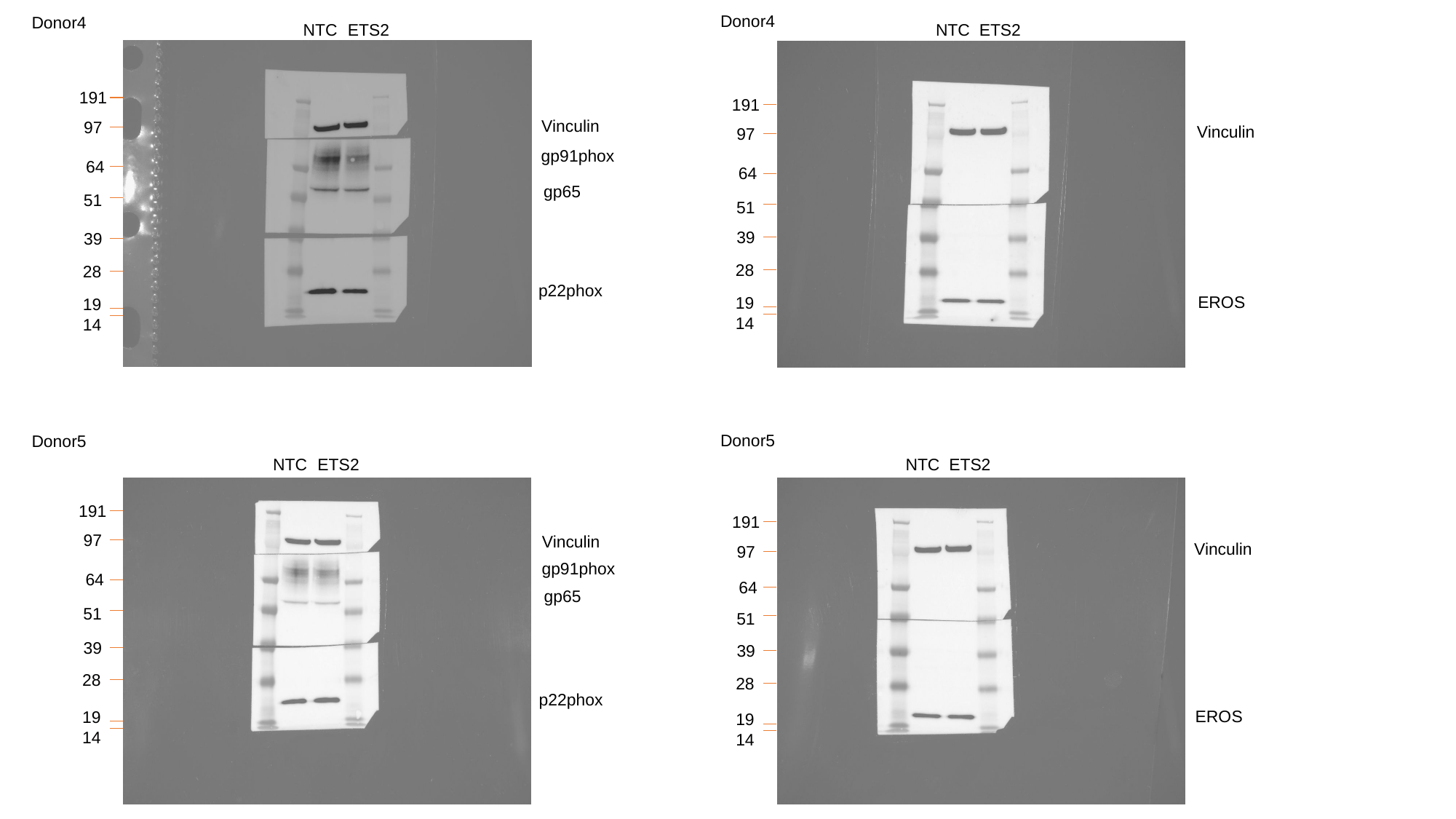

Donor4
Donor4
NTC
ETS2
NTC
ETS2
191
191
Vinculin
97
Vinculin
97
gp91phox
64
64
gp65
51
51
39
39
28
28
p22phox
EROS
19
19
14
14
Donor5
Donor5
NTC
ETS2
NTC
ETS2
191
191
97
Vinculin
Vinculin
97
gp91phox
64
64
gp65
51
51
39
39
28
28
p22phox
EROS
19
19
14
14

## Slide 4
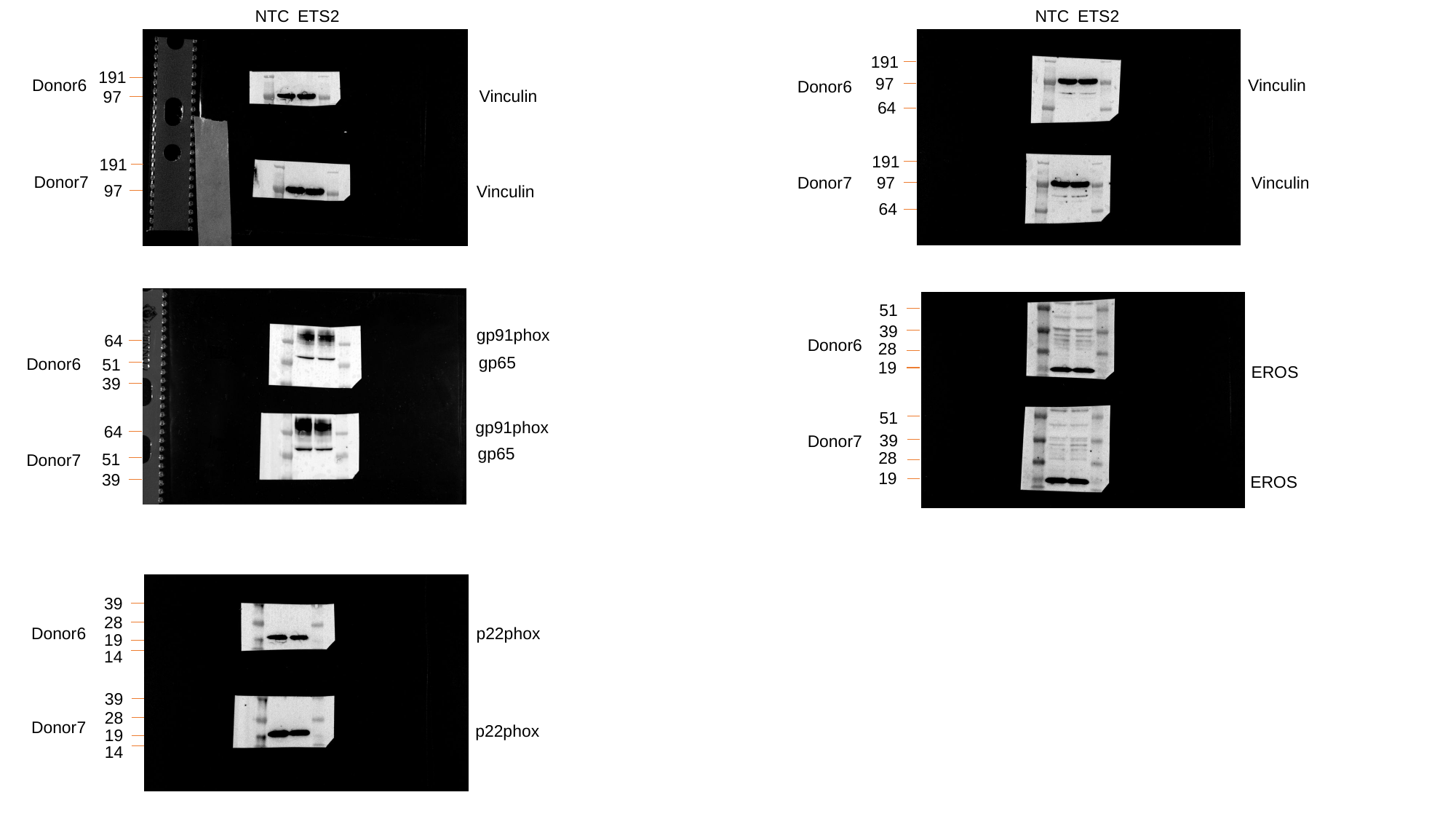

NTC
ETS2
NTC
ETS2
191
191
97
Vinculin
Donor6
Donor6
Vinculin
97
64
191
191
Donor7
Donor7
97
Vinculin
97
Vinculin
64
51
39
gp91phox
64
Donor6
28
gp65
Donor6
51
19
EROS
39
51
gp91phox
64
39
Donor7
gp65
28
51
Donor7
19
39
EROS
39
28
Donor6
p22phox
19
14
39
28
Donor7
p22phox
19
14

## Slide 5
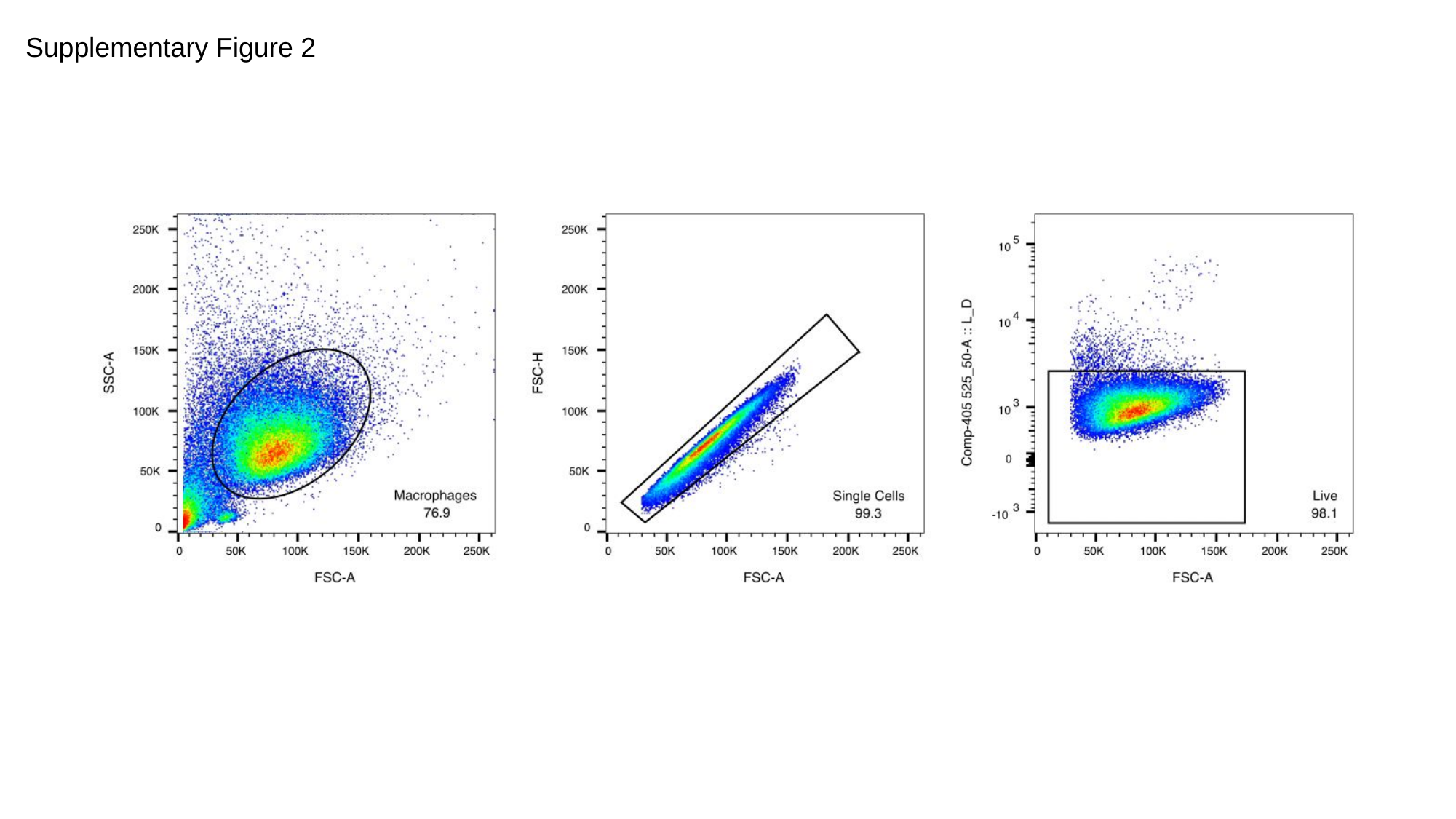

Supplementary Figure 2
