# Supplementary material for: A disease-associated gene desert directs macrophage inflammation through ETS2
Source: Nature. 2024 Jun 5;630(8016):447–56. doi: 10.1038/s41586-024-07501-1 (PMC11168933; doi:10.1038/s41586-024-07501-1)
Supplement: Supplementary file 2 — Reporting Summary [file 41586_2024_7501_MOESM2_ESM.pdf]

nature portfolio

Corresponding author(s): James Lee
Last updated by author(s): Feb 29, 2024

# Reporting Summary

Nature Portfolio wishes to improve the reproducibility of the work that we publish. This form provides structure for consistency and transparency in reporting. For further information on Nature Portfolio policies, see our Editorial Policies and the Editorial Policy Checklist.

## Statistics

For all statistical analyses, confirm that the following items are present in the figure legend, table legend, main text, or Methods section.

| n/a | Confirmed | |
|---|---|---|
| ☐ | ☒ | The exact sample size (*n*) for each experimental group/condition, given as a discrete number and unit of measurement |
| ☐ | ☒ | A statement on whether measurements were taken from distinct samples or whether the same sample was measured repeatedly |
| ☐ | ☒ | The statistical test(s) used AND whether they are one- or two-sided<br>*Only common tests should be described solely by name; describe more complex techniques in the Methods section.* |
| ☐ | ☒ | A description of all covariates tested |
| ☐ | ☒ | A description of any assumptions or corrections, such as tests of normality and adjustment for multiple comparisons |
| ☐ | ☒ | A full description of the statistical parameters including central tendency (e.g. means) or other basic estimates (e.g. regression coefficient) AND variation (e.g. standard deviation) or associated estimates of uncertainty (e.g. confidence intervals) |
| ☐ | ☒ | For null hypothesis testing, the test statistic (e.g. *F*, *t*, *r*) with confidence intervals, effect sizes, degrees of freedom and *P* value noted<br>*Give P values as exact values whenever suitable.* |
| ☐ | ☒ | For Bayesian analysis, information on the choice of priors and Markov chain Monte Carlo settings |
| ☒ | ☐ | For hierarchical and complex designs, identification of the appropriate level for tests and full reporting of outcomes |
| ☐ | ☒ | Estimates of effect sizes (e.g. Cohen's *d*, Pearson's *r*), indicating how they were calculated |

*Our web collection on statistics for biologists contains articles on many of the points above.*

## Software and code

Policy information about availability of computer code

| | |
|---|---|
| Data collection | FACS Diva v.6.0 was used to collect flow cytometry data. Next-generation sequencing data was obtained using in-built software on Illumina Sequencers and demultiplexed and converted to FASTQ files using bcl2fastq (v.1.8.4) or bcl2fastq2 (v.2.20). U-PLEX data was obtained using DISCOVERY WORKBENCH Software (v.4.0). NanoString CosMx SMI was used to collect spatial transcriptomics data. |
| Data analysis | Code to reproduce analyses are available at https://github.com/JamesLeeLab/chr21q22_manuscript, https://github.com/chr1swallace/ibd-ets2-analysis, and https://github.com/qzhang314/PRS_IBD_subpheno. Flow cytometry data was gated with FlowJo v10.8.1. Metabolomic GC-MS analysis was performed using MANIC (v.3.0), an adaptation of GAVIN. Densitometry analysis was performed using ImageJ (v1.53g). Next-generation sequencing data was pre-processed using FastQC (v.0.11.8), MultiQC (v.0.9), Trim Galore (v.0.6.0), Picard (v.2.1.1), deepTools (v.3.3.1), BBSplit (from BBMap v.36.20), Burrows-Wheeler Aligner (ChIP-seq, ATC-seq) or Bowtie2 (v.2.4.4; CUT&RUN) or HISAT2 (v.2.1.0; RNA-seq), Rsubread (v.2.12.3), and splitSNP (ATAC-seq). Polygenic risk score analysis was run using Plink (v.1.9). Spatial transcriptomics data were pre-processed using the AoMx Spatial Informatics Platform (NanoString Technologies). The following R (v.4.2.1) packages were used in this work: fine-mapping: susieR (v.0.12.35), ssimp (v.0.5.6), co-localisation: coloc (v.5.2.0), allele-specific binding: BaalChIP (v.1.12.0), gene-set variation analysis: GSVA (v.1.44.5), gene-set enrichment analysis: fgsea (v.1.23.2), differential expression analysis: limma (v.3.52.4), irreproducible discovery rate: idr (v.1.3), GenomicRanges (v.1.48.0), MPRA analysis: QuASAR (v.0.1) and les (v.1.46.0), co-expression analysis: Hmisc (v5.0.1), single cell RNA-seq: Seurat (v.4), spatial transcriptomics: InsituType, plots were made using EnhancedVolcano (v.1.14.0), EnhancedHeatmap (v.1.26.0), ggplot2 (v.3.4.0), gplots (v.3.1.3), karyoploteR (v.1.22.0). The following Python packages were used: peak-calling: MACS2 (v.2.2.5), genetic enrichment: SNPsea (v.1.0.3), Rank-Ordering of Super-Enhancers: ROSE, ChIP-seq pre-processing: SAMtools (v.1.11), spatial transcriptomics: napari (v0.4.17). |

For manuscripts utilizing custom algorithms or software that are central to the research but not yet described in published literature, software must be made available to editors and reviewers. We strongly encourage code deposition in a community repository (e.g. GitHub). See the Nature Portfolio guidelines for submitting code & software for further information.

## Data

Policy information about availability of data

All manuscripts must include a data availability statement. This statement should provide the following information, where applicable:

- Accession codes, unique identifiers, or web links for publicly available datasets
- A description of any restrictions on data availability
- For clinical datasets or third party data, please ensure that the statement adheres to our policy

The datasets produced in this study are accessible via the following repositories:
MPRA (GEO: GSE229472)
RNA-seq of ETS2 or chr21q22-edited TPP macrophages (EGA: EGAD00001011338)
RNA-seq of ETS2 overexpression (EGA: EGAD00001011341)
RNA-seq of MEK inhibitor-treated TPP macrophages (EGA: EGAD00001011337)
H3K27ac ChIP-seq in TPP macrophages (EGA: EGAD00001011351)
ATAC-seq and H3K27ac ChIP-seq in ETS2-overexpressing or -edited macrophages (EGA: EGAD50000000154)
ETS2 CUT&RUN (EGA: EGAD00001011349)
Biopsy RNA-seq data (EGA: EGAD00001011333)
MetaboLights: Metabolomics (MTBLS7665)
The phenotype and genotype data used for the PRS analysis are available upon application to the IBD Bioresource (https://www.ibdbioresource.nihr.ac.uk/).

## Research involving human participants, their data, or biological material

Policy information about studies with human participants or human data. See also policy information about sex, gender (identity/presentation), and sexual orientation and race, ethnicity and racism.

| | |
|---|---|
| Reporting on sex and gender | Information on sex and gender on leukocyte cones were anonymised at the point of collection and not provided to the research team. Sex information was provided for inflammatory bowel disease patient samples for matching. |
| Reporting on race, ethnicity, or other socially relevant groupings | We do not report on race, ethnicity, or other socially relevant groupings. |
| Population characteristics | Population characteristics of human research participants were not used as covariates. |
| Recruitment | Leukocyte apheresis cones were collected by NHS Blood and Transplant from healthy platelet donors. Patients with active inflammatory bowel disease, who were not receiving immunosuppressive or biologic therapies, were recruited by clinicians at the Royal Free Hospital London. Inflammatory bowel disease patients who were homozygous for either the rs2836882 risk or non-risk allele were recruited via the NIHR BioResource. |
| Ethics oversight | Ethical approval to obtain blood from healthy individuals and samples from inflammatory bowel disease patients was provided by the London - Brent Regional Ethics Committee (REC: 21/LO/0682). Liver samples were provided by the Tissue Access for Patient Benefit (TAP-B, part of the UCL-RFH BioBank) with approval from the Wales Research Ethics Committee 4 (REC 16/WA/0289). |

Note that full information on the approval of the study protocol must also be provided in the manuscript.

# Field-specific reporting

Please select the one below that is the best fit for your research. If you are not sure, read the appropriate sections before making your selection.

☒ Life sciences ☐ Behavioural & social sciences ☐ Ecological, evolutionary & environmental sciences

For a reference copy of the document with all sections, see nature.com/documents/nr-reporting-summary-flat.pdf

# Life sciences study design

All studies must disclose on these points even when the disclosure is negative.

| | |
|---|---|
| Sample size | Where relevant, sample sizes were pre-determined using power calculations based on anticipated effect sizes and variability observed in previous similar experiments. Sample size is stated in each panel and in the Methods. |
| Data exclusions | Data were only excluded if they failed pre-defined quality control metrics or if the sample had insufficient material to perform the experiment (applied to one biopsy RNA-seq sample). Otherwise results represent all data points collected from the experiments indicated. |
| Replication | The number of biological replicates for individual experiments are stated in figure legends and methods. Technical replicates were summarised before statistical analyses. All experimental findings were reproducible - as indicated by the statistical analysis described. |

| | |
|---|---|
| Randomization | Biopsy culture experiments were performed by randomly allocating 2 biopsies to each experimental condition. H3K27ac ChIP-seq in minor and major allele homozygous samples and RT-qPCR in inflammatory bowel disease patient samples could not be randomised due to genotyping. All other experiments included cells from the same donor collected at the same time in different experimental groups e.g. transfection of non-targeting control RNPs for CRISPR, reverse complement ETS2 for overexpression, IgG pulldowns for CUT&RUN, vehicle controls for MEK inhibitor samples. |
| Blinding | Blinding was not performed as all experiments were based on objective quantitative measurements (e.g. flow cytometry, CUT&RUN, RNA-seq) and analyses were automated and applied equally to all samples. |

# Reporting for specific materials, systems and methods

We require information from authors about some types of materials, experimental systems and methods used in many studies. Here, indicate whether each material, system or method listed is relevant to your study. If you are not sure if a list item applies to your research, read the appropriate section before selecting a response.

## Materials & experimental systems

| n/a | Involved in the study |
|---|---|
| ☐ | ☒ Antibodies |
| ☒ | ☐ Eukaryotic cell lines |
| ☐ | ☒ Palaeontology and archaeology |
| ☒ | ☐ Animals and other organisms |
| ☒ | ☐ Clinical data |
| ☒ | ☐ Dual use research of concern |
| ☒ | ☐ Plants |

## Methods

| n/a | Involved in the study |
|---|---|
| ☐ | ☒ ChIP-seq |
| ☐ | ☒ Flow cytometry |
| ☒ | ☐ MRI-based neuroimaging |

## Antibodies

Antibodies used

PU.1 antibody, #2266S, Cell Signaling
anti-H3K27ac antibody, ab4729, Abcam
Recombinant rabbit IgG isotype control, ab172730, clone EPR25A, Abcam
gp91phox antibody, sc-130543, Santa Cruz
p22phox antibody, sc-20781, Santa Cruz
anti-C17ORF62/EROS antibody, HPA045696, Atlas Antibodies
anti-vinculin, V4505, Sigma
goat anti-mouse IgG-horseradish peroxidase, 115-036-071, Jackson Immuno
goat anti-rabbit IgG-horseradish peroxidase, 111-036-046, Jackson Immuno
ETS2 antibody, PA528053, ThermoFisher
rabbit IgG isotype control, #66362, clone DA1E, Cell Signaling
PE/Dazzle 594 CD11b antibody, #301347, clone ICRF44, BioLegend
evolve405 CD14 antibody, #83-0149-42, clone 61D3, ThermoFisher
PerCP CD16 antibody, #302029, clone 3G8, BioLegend
FITC CD68 antibody, #333805, clone Y1/82A, BioLegend

Validation

PU.1 antibody, #2266S, Cell Signaling: validated for ChIP-sequencing: https://www.cellsignal.com/products/primary-antibodies/pu-1-antibody/2266
anti-H3K27ac antibody, Ab4729, Abcam: validated for ChIP-sequencing: https://www.abcam.com/products/primary-antibodies/histone-h3-acetyl-k27-antibody-chip-grade-ab4729.html
Recombinant rabbit IgG isotype control, ab172730, Abcam: validated for ChIP-sequencing: https://www.abcam.com/products/primary-antibodies/rabbit-igg-monoclonal-epr25a-isotype-control-ab172730.html
gp91phox antibody, sc-130543, Santa Cruz: validated for western blot: https://datasheets.scbt.com/sc-130543.pdf
p22phox antibody, sc-20781, Santa Cruz: validated for western blot: https://datasheets.scbt.com/sc-20781.pdf
anti-C17ORF62/EROS antibody, HPA045696, Atlas Antibodies: previously validated to bind to EROS in PMID: 36421765
anti-vinculin, V4505, Sigma: validated for western blot: https://www.sigmaaldrich.com/GB/en/product/sigma/v4505
goat anti-mouse IgG-horseradish peroxidase, 115-036-071, Jackson Immuno - validated for western blot: https://www.jacksonimmuno.com/catalog/products/115-036-071
goat anti-rabbit IgG-horseradish peroxidase, 111-036-046, Jackson Immuno - validated for western blot: https://www.jacksonimmuno.com/catalog/products/111-036-046
ETS2 antibody, PA528053, ThermoFisher: validated for Western blot: https://www.thermofisher.com/antibody/product/ETS2-Antibody-Polyclonal/PA5-28053; produced reproducible CUT&RUN peaks that met acceptable quality metrics
rabbit IgG isotype control, #66362, Cell Signaling; validated for CUT&RUN: https://www.cellsignal.com/products/primary-antibodies/rabbit-da1e-mab-igg-xp-isotype-control-cut-amp-run/66362
PE/Dazzle 594 CD11b antibody, #301347, BioLegend: validated for flow cytometry: https://www.biolegend.com/en-gb/explore-new-products/pe-dazzle-594-anti-human-cd11b-antibody-10195
evolve605 CD14 antibody, #83-0149-42, ThermoFisher: validated for flow cytometry in PMID: 28813661
PerCP CD16 antibody, #302029, BioLegend: validated for flow cytometry; https://www.biolegend.com/de-at/products/percp-anti-human-cd16-antibody-4340
FITC CD68 antibody, #333805, BioLegend: validated for flow cytometry: https://www.biolegend.com/nl-be/products/fitc-anti-human-cd68-antibody-4844

## Palaeontology and Archaeology

| | |
|---|---|
| Specimen provenance | Genotypes at the disease-associated chr21q22 candidate SNPs were determined using publicly available genomes from seven Neanderthal individuals, one Denisovan individual, and one Neanderthal and Denisovan F1 individual. |
| Specimen deposition | Publicly available genomes. |
| Dating methods | No new dates are provided. |

☒ Tick this box to confirm that the raw and calibrated dates are available in the paper or in Supplementary Information.

| | |
|---|---|
| Ethics oversight | Because the genomes were publicly available, no ethical approval or guidance was necessary. |

Note that full information on the approval of the study protocol must also be provided in the manuscript.

## Plants

| | |
|---|---|
| Seed stocks | *Report on the source of all seed stocks or other plant material used. If applicable, state the seed stock centre and catalogue number. If plant specimens were collected from the field, describe the collection location, date and sampling procedures.* |
| Novel plant genotypes | *Describe the methods by which all novel plant genotypes were produced. This includes those generated by transgenic approaches, gene editing, chemical/radiation-based mutagenesis and hybridization. For transgenic lines, describe the transformation method, the number of independent lines analyzed and the generation upon which experiments were performed. For gene-edited lines, describe the editor used, the endogenous sequence targeted for editing, the targeting guide RNA sequence (if applicable) and how the editor was applied.* |
| Authentication | *Describe any authentication procedures for each seed stock used or novel genotype generated. Describe any experiments used to assess the effect of a mutation and, where applicable, how potential secondary effects (e.g. second site T-DNA insertions, mosiacism, off-target gene editing) were examined.* |

## ChIP-seq

### Data deposition

☒ Confirm that both raw and final processed data have been deposited in a public database such as GEO.

☒ Confirm that you have deposited or provided access to graph files (e.g. BED files) for the called peaks.

| | |
|---|---|
| Data access links<br>*May remain private before publication.* | Data have been deposited in EGA:<br>H3K27ac ChIP-seq in TPP macrophages (EGA: EGAD00001011351)<br>H3K27ac ChIP-seq in ETS2-overexpressing or -edited macrophages (EGA: EGAD50000000154)<br>ETS2 CUT&RUN (EGA: EGAD00001011349) |
| Files in database submission | H3K27ac ChIP-seq in TPP macrophages<br>- H3K27ac ChIP, rs2836882 MajorAlleleHom, rep1<br>- H3K27ac ChIP, rs2836882 MajorAlleleHom, rep2<br>- H3K27ac ChIP, rs2836882 MinorAlleleHom, rep1<br>- H3K27ac ChIP, rs2836882 MinorAlleleHom, rep2<br>- Input DNA, rs2836882 MajorAlleleHom, rep1<br>- Input DNA, rs2836882 MajorAlleleHom, rep2<br>- Input DNA, rs2836882 MinorAlleleHom, rep1<br>- Input DNA, rs2836882 MinorAlleleHom, rep2<br><br>ETS2 CUT&RUN in TPP macrophages<br>- TPP_donor1, ETS2 CUT&RUN<br>- TPP_donor1_IgG control CUT&RUN<br>- TPP_donor2_ETS2 CUT&RUN<br>- TPP_donor2_IgG control CUT&RUN<br><br>H3K27ac ChIP-seq in ETS2-overexpressing or ETS2-disrupted TPP macrophages<br>- H3K27ac ChIP, ETS2 500 ng, donor1<br>- H3K27ac ChIP, ETS2 REV 500 ng, donor1<br>- H3K27ac ChIP ETS2 500 ng, donor2<br>- H3K27ac ChIP ETS2 REV 500 ng, donor2<br>- H3K27ac ChIP ETS2 500 ng, donor3<br>- H3K27ac ChIP ETS2 REV 500 ng, donor3<br>- Input DNA, ETS2 500 ng, donor1<br>- Input DNA, ETS2 REV 500 ng, donor1<br>- Input DNA, ETS2 500 ng, donor2<br>- Input DNA, ETS2 REV 500 ng, donor2 |

- Input DNA, ETS2 500 ng, donor3
- Input DNA, ETS2 REV 500 ng, donor3
- H3K27ac ChIP, NTC, donor1
- H3K27ac ChIP, ETS2KO, donor1
- H3K27ac ChIP, NTC, donor2
- H3K27ac ChIP, ETS2KO, donor2
- H3K27ac ChIP, NTC, donor3
- H3K27ac ChIP, ETS2KO, donor3
- Input DNA, NTC, donor1
- Input DNA, ETS2KO, donor1
- Input DNA, NTC, donor2
- Input DNA, ETS2KO, donor2
- Input DNA, NTC, donor3
- Input DNA, ETS2KO, donor3

Genome browser session
(e.g. UCSC)

IGV session including tracks from ETS2 CUT&RUN, ATAC-seq in TPP macrophages, and H3K27ac ChIP-seq in TPP macrophages from rs2836882 minor and major allele homozygotes available at https://tinyurl.com/23g9h3bn

## Methodology

Replicates

Biological replicates (healthy donors) for H3K27ac ChIP:
2 replicates: rs2836882 homozygote major (H3K27ac ChIP-seq and Input)
2 replicates: rs2836882 homozygote minor (H3K27ac ChIP-seq and Input)

Biological replicates (healthy donors) for ETS2 CUT&RUN:
2 replicates for ETS2 CUT&RUN in TPP macrophages with corresponding IgG controls

Biological replicates (healthy donors) for H3K27ac ChIP-seq in ETS2-overexpressing or ETS2-disrupted TPP macrophages:
3 replicates: ETS2 500 ng and REV ETS2 500 ng (H3K27ac ChIP-seq and Input)
3 replicates: NTC and ETS2 KO (H3K27ac ChIP-seq and Input)

Sequencing depth

H3K27ac ChIP. All reads are single-end, 50bp in length.

| Sample | Read depth (M) | Uniquely mapped reads |
|---|---|---|
| - H3K27ac ChIP, MajorAlleleHom, rep1 | 28.9 | 16336754 |
| - H3K27ac ChIP, MajorAlleleHom, rep2 | 30.7 | 20058550 |
| - H3K27ac ChIP, MinorAlleleHom, rep1 | 27.4 | 15622745 |
| - H3K27ac ChIP, MinorAlleleHom, rep2 | 24.9 | 16204315 |
| - Input DNA, MajorAlleleHom, rep1 | 21.4 | 12576448 |
| - Input DNA, MajorAlleleHom, rep2 | 22 | 14679612 |
| - Input DNA, MinorAlleleHom, rep1 | 26.6 | 14253281 |
| - Input DNA, MinorAlleleHom, rep2 | 25.5 | 16069327 |

ETS2 CUT&RUN. All reads are paired-end, 100bp in length.

| Sample | Read depth | Read Pair Unique | Read Pair Not Optical Duplicates |
|---|---|---|---|
| - TPP_donor1, ETS2 CUT&RUN | 44,822,163 | 23,406,807 | 6,269,062 |
| - TPP_donor1_IgG control CUT&RUN | 46,996,015 | 17,294,478 | 5,400,929 |
| - TPP_donor2_ETS2 CUT&RUN | 50,848,338 | 20,508,435 | 6,612,914 |
| - TPP_donor2_IgG control CUT&RUN | 37,626,720 | 14,520,763 | 4,443,194 |

H3K27ac ChIP-seq in ETS2-overexpressing or ETS2-disrupted TPP macrophages. All reads are paired-end, 100bp in length.

| Sample | Uniquely mapped read pairs |
|---|---|
| H3K27ac_TPP_D1_ETS2_500 | 62821646 |
| H3K27ac_TPP_D1_REV_500 | 54400167 |
| H3K27ac_TPP_D1_ETS2input_500 | 62539567 |
| H3K27ac_TPP_D1_REVinput_500 | 55850854 |
| H3K27ac_TPP_D2_ETS2_500 | 59509120 |
| H3K27ac_TPP_D2_REV_500 | 57390645 |
| H3K27ac_TPP_D2_ETS2input_500 | 59945178 |
| H3K27ac_TPP_D2_REVinput_500 | 58958544 |
| H3K27ac_TPP_D3_ETS2_500 | 59798070 |
| H3K27ac_TPP_D3_REV_500 | 59256158 |
| H3K27ac_TPP_D3_ETS2input_500 | 54136028 |
| H3K27ac_TPP_D3_REVinput_500 | 54702503 |
| H3K27ac_TPP_NCI106NTC | 58039063 |
| H3K27ac_TPP_NCI106KO | 58709735 |
| H3K27ac_TPP_NCI106NTC_input | 57860350 |
| H3K27ac_TPP_NCI106KO_input | 65846125 |
| H3K27ac_TPP_NCI107NTC | 58746162 |
| H3K27ac_TPP_NCI107KO | 58965593 |
| H3K27ac_TPP_NCI107NTC_input | 64677003 |
| H3K27ac_TPP_NCI107KO_input | 58014156 |
| H3K27ac_TPP_NCI109NTC | 67028007 |
| H3K27ac_TPP_NCI109KO | 59109486 |
| H3K27ac_TPP_NCI109NTC_input | 61411094 |
| H3K27ac_TPP_NCI109KO_input | 59967525 |

Antibodies

anti-H3K27ac antibody, ab4729, abcam
rabbit IgG, ab172730, abcam

anti-ETS2 antibody, PA528053, ThermoFisher Scientific
Rabbit (DA1E) mAb IgG XP® Isotype Control, #66362, Cell Signaling Technology

Peak calling parameters

H3K27ac ChIP-seq:
macs2 callpeak -t sample1.bam -c input1.bed -g hs -n H3k27ac_1_hg19 -f AUTO --outdir PEAKS --qvalue 0.01 -B --nomodel --extsize=200

ETS2 CUT&RUN:
- initial peak calling: macs2 callpeak -t sample1.bam -c IgG1.bam -g hs -n CRETS2_Ther1_hg19 -f BAMPE --keep-dup all --qvalue 0.05 -B --nomodel
- idr on peak calls: mu = 3, sigma = 1, rho = 0.9, eps = 0.001, p = .5

Full code on GIthub (see below)

Data quality

H3K27ac ChIP-seq:
The data were trimmed with Trimgalore, with parameters: --phred33 -q 24 --illumina --length 30.
Unmapped , multi-mapped, chimeric and duplicate reads were excluded using Samtools, with parameters: -b -h -F 4 -F 256 -F 1024 -F 2048 -q 15.
NSC and RSC values were obtained using phantompeakqualtools, with the SPP R package.
Peaks were called using a FDR threshold of 1% (cf. above).

| Sample | Number of peaks (FDR 1%) | Above 5-fold enrichment | NSC | RSC |
|---|---|---|---|---|
| H3K27ac, ChIP, MajorAlleleHom, rep1 | 40114 | 38820 | 1.3 | 1.1 |
| H3K27ac, ChIP, MajorAlleleHom, rep2 | 14604 | 14544 | 1.05 | 0.8 |
| H3K27ac, ChIP, MinorAlleleHom, rep1 | 34498 | 33594 | 1.3 | 1.1 |
| H3K27ac, ChIP, MinorAlleleHom, rep2 | 36419 | 36046 | 1.4 | 1.1 |

ETS2 CUT&RUN:
The data were trimmed with Trimgalore, with parameters: --phred33 -q 24 --illumina --length 25 --paired --stringency 6.
Reads were aligned with Bowtie2 following the guidelines by Skene & Henikoff (2017, Elife), with parameters: --local --very-sensitive-local --no-mixed --no-discordant --phred33 -I 10 -X 700.
Unmapped , multi-mapped and chimeric reads were excluded using Samtools, with parameters: -b -h -F 4 -F 256 -F 2048 -q 15.
Peaks were called using a FDR threshold of 5% (cf. above) and IDR analysis was performed with a 1% cut-off (cf. main text and methods) to select the reproducible peaks between the 2 biological replicates.

| Sample | Number of peaks (FDR 5%) | Above 5-fold enrichment |
|---|---|---|
| TPP_donor1, ETS2 CUT&RUN | 46,813 | 38,183 |
| TPP_donor2_ETS2 CUT&RUN | 58,918 | 42,395 |

Irreproducible discovery rate: 6,560 reproducible peaks

H3K27ac ChIP-seq in ETS2-overexpressing or ETS2-disrupted TPP macrophages.
Data processed as described above.

| Sample | Number of peaks (FDR 1%) | Above 5-fold enrichment |
|---|---|---|
| H3K27ac_TPP_D1_ETS2_500 | 38930 | 18265 |
| H3K27ac_TPP_D1_REV_500 | 39781 | 16573 |
| H3K27ac_TPP_D2_ETS2_500 | 38915 | 15639 |
| H3K27ac_TPP_D2_REV_500 | 35716 | 13646 |
| H3K27ac_TPP_D3_ETS2_500 | 32279 | 14776 |
| H3K27ac_TPP_D3_REV_500 | 31847 | 15266 |
| H3K27ac_TPP_NCI106NTC | 50712 | 23417 |
| H3K27ac_TPP_NCI106KO | 48879 | 20446 |
| H3K27ac_TPP_NCI107NTC | 58789 | 25190 |
| H3K27ac_TPP_NCI107KO | 48588 | 23760 |
| H3K27ac_TPP_NCI109NTC | 53762 | 23990 |
| H3K27ac_TPP_NCI109KO | 52799 | 25044 |

Software

All code and software details are available at https://github.com/JamesLeeLab/chr21q22_manuscript/tree/main/ChIP-seq and https://github.com/JamesLeeLab/chr21q22_manuscript/tree/main/CUT%26RUN. Also deposited at https://zenodo.org/records/10707942.

# Flow Cytometry

## Plots

Confirm that:

☒ The axis labels state the marker and fluorochrome used (e.g. CD4-FITC).

☒ The axis scales are clearly visible. Include numbers along axes only for bottom left plot of group (a 'group' is an analysis of identical markers).

☒ All plots are contour plots with outliers or pseudocolor plots.

☒ A numerical value for number of cells or percentage (with statistics) is provided.

## Methodology

| | |
|---|---|
| Sample preparation | Monocytes were positively selected from leukocyte cones using CD14 Microbeads. Macrophage differentiation was performed using conditions that model chronic inflammation (TPP): 3 days GM-CSF (50ng/mL) followed by 3 days GM-CSF, TNFa (50ng/mL), PGE2 (1mg/mL), and Pam3CSK4 (1mg/mL).<br><br>RNA abundance was quantified by PrimeFlow (ThermoFisher) in TPP macrophages on days 0, 3, 4, 5, and 6 of TPP differentiation. Target probes specific for ETS2 (Alexa Fluor 647), BRWD1 (Alexa Fluor 568) and PSMG1 (Alexa Fluor 568) were used according to the manufacturer's instructions.<br><br>For assessment of myeloid marker expression, macrophages were detached with Accutase on day 6 of culture and were stained with CD11b PE/Dazzle 594, CD14 evolve405, CD16 PerCP, and CD68 FITC, along with Live/Dead Fixable Aqua Dead Cell Stain (ThermoFisher) and Fc Receptor Blocking Reagent (Miltenyi).<br><br>Phagocytosis was assayed by quantifying uptake of uptake of fluorescently-labelled Zymosan particles (Green Zymosan, Abcam) according to the manufacturer's instructions. Cells were stained with Live/Dead Fixable Aqua Dead Cell Stain (ThermoFisher) prior to flow cytometry. |
| Instrument | BD LSRFortessa X-20 |
| Software | FACS Diva was used to collect flow cytometry data. FlowJo v10 was used to for data analysis. |
| Cell population abundance | N/A - no sorting was performed |
| Gating strategy | Macrophages were gated by FSC-A/SSC-A and singlets were gated by FSC-A/FSC-H. Live cells were gated (and viability was quantified) using Live/Dead Fixable Aqua Dead Cell Stain. |

☒ Tick this box to confirm that a figure exemplifying the gating strategy is provided in the Supplementary Information.
